# Stroke Prevention in Atrial Fibrillation: Focus on Latin America

**DOI:** 10.5935/abc.20160116

**Published:** 2016-12

**Authors:** Ayrton R. Massaro, Gregory Y. H. Lippp

**Affiliations:** 1 Hospital Sírio Libanês, São Paulo, SP - Brazil; 2 Divisão de Neurologia - Instituto do Cérebro do Rio Grande do Sul - PUCRS, Porto Alegre, RS - Brazil; 3 University of Birmingham Institute of Cardiovascular Sciences - City Hospital - Birmingham, United Kingdom; 4 Aalborg Thrombosis Research Unit - Department of Clinical Medicine - Faculty of Health - Aalborg University, Aalborg, Denmark

**Keywords:** Atrial Fibrillation, Prevention, Stroke, Latin America, Anticoagulants

## Abstract

Atrial fibrillation (AF) is the most common sustained cardiac arrhythmia, with an estimated prevalence of 1-2% in North America and Europe. The increased prevalence of AF in Latin America is associated with an ageing general population, along with poor control of key risk factors, including hypertension. As a result, stroke prevalence and associated mortality have increased dramatically in the region. Therefore, the need for effective anticoagulation strategies in Latin America is clear. The aim of this review is to provide a contemporary overview of anticoagulants for stroke prevention.

The use of vitamin K antagonists (VKAs, eg, warfarin) and aspirin in the prevention of stroke in patients with AF in Latin America remains common, although around one fifth of all AF patients receive no anticoagulation. Warfarin use is complicated by a lack of access to effective monitoring services coupled with an unpredictable pharmacokinetic profile. The overuse of aspirin is associated with significant bleeding risks and reduced efficacy for stroke prevention in this patient group. The non-VKA oral anticoagulants (NOACbs) represent a potential means of overcoming many limitations associated with VKA and aspirin use, including a reduction in the need for monitoring and a reduced risk of hemorrhagic events.

The ultimate decision of which anticoagulant drug to utilize in AF patients depends on a multitude of factors. More research is needed to appreciate the impact of these factors in the Latin American population and thereby reduce the burden of AF-associated stroke in this region.

## Introduction

Atrial fibrillation (AF) is the most common sustained cardiac arrhythmia with an estimated prevalence of 1-2% in North American and European countries.^1^ However, the prevalence of AF in Latin America is largely unknown due to a paucity of research in this region.^2^ As a result of the emergence of cardiovascular disease and risk factors in this region, it is thought that AF is a major problem with an estimated 1.5 million patients affected in Brazil and 230,000 patients affected in Venezuela, a figure estimated to rise to 1 million by the year 2050.^3^ Therefore, it would appear as though AF is a common clinical phenomenon, with a rising prevalence in many nations in Latin America.

The incidence of stroke is significantly increased in patients diagnosed with AF, with some data suggesting up to a fivefold increase in stroke risk with AF directly responsible for an increasing percentage of ischemic strokes with increasing age in the elderly population.^4,5^ The World Health Organisation (WHO) estimated that 1.9 million people survived a stroke in Latin America in 2004, with almost one quarter of those experiencing a first time stroke.^6^ It is estimated that deaths due to stroke are set to at least double by the year 2024 in Latin America.^7^ Recent epidemiological data suggests that over 700,000 incident strokes were recorded in Brazil alone in 2010, with over 141,000 deaths attributed to stroke in 2010 alone.^7^ These figures represent an approximate twofold increase on statistics published in 1990, a trend seen in many Latin American nations.^7^

Strokes related to AF are considered more severe than non-AF strokes due to the large infarction size associated with occlusion of the proximal middle cerebral artery and are accompanied by a greater risk of in-hospital death, and increased risk of recurrent stroke.^8^ Therefore, AF-related stroke appears to be an important problem in Latin America, particularly as risk factors for AF are often poorly controlled in this population.^8^ In combination with the association of AF with other co-morbidities and all-cause mortality, AF is a significant public health burden in the region that will have deep implications for public health practice in the future.^6^

The use of oral anticoagulants as a prophylactic treatment for those at increased risk of thromboembolism is key for the prevention of stroke in patients with AF.^9^ Vitamin K antagonists (VKAs), including warfarin, remain one of the most widely used approaches for stroke prevention in non-valvular AF and have an established high level of efficacy; adjusted dose warfarin therapy reduces the risk of ischemic stroke by 64% and all-cause mortality by 26%.^10^ However warfarin use requires optimal anticoagulation control, defined as a mean individual time in therapeutic range (TTR) > 70%, which is associated with best efficacy and safety outcomes.^11^

The common use of aspirin is also observed in many patients, despite evidence that it is substantially less effective than warfarin at stroke prevention and is associated with a similar level of major bleeding risk.^12^ The introduction of non-VKA oral anticoagulants (NOACs), including direct thrombin inhibitors and factor Xa inhibitors, has provided physicians with an alternative method of preventing stroke in patients with AF, which may overcome many of the limitations of VKAs and aspirin use.^13,14^ The NOACs have a predictable pharmacokinetic profile and do not require regular anticoagulant monitoring.^15^ Furthermore, these agents have been shown to be non-inferior to warfarin in clinical stroke prevention studies, prompting their inclusion in North American and European guidelines.^15,16^

Despite the evidence in support of NOAC use in routine stroke prevention for patients with AF, the influence of individual patient factors on the choice of anticoagulant needs to be considered.^17^ The clinical decision-making process remains sensitive to local health care needs and resources, including the prevalence of co-morbidities and availability of medications or monitoring facilities. Therefore, the selection of appropriate therapeutic agents for stroke prevention in patients with AF in Latin America is a multi-factorial issue.

The aim of this review is to provide an update to physicians on current best practice in stroke prevention in AF. This will include an overview of contemporary evidence for the use of NOACs, while specifically addressing the challenges inherent in delivering optimal care in the context of Latin America.

## Methods

A comprehensive review of the literature was performed in order to achieve the objective for this review. Database searches were conducted using online resources, including the following key words in combination: NOAC, warfarin, aspiring, stroke, and atrial fibrillation. Data from papers published from January 2005 to April 2015 were included in order to maintain a contemporary perspective on the clinical issue. Papers focusing on the context of stroke prevention associated with AF in Latin America were specifically sought, while a wider discussion of the health needs of this population is also considered to provide context to health decision-making processes in the region.

### Overview of stroke epidemiology in Latin America

Latin American nations are experiencing a period of rapid economic growth.^18^ This phase of growth has been characterized by uneven socioeconomic changes, leading to significant effects on lifestyle and demographic indicators of health.^18^ Lifestyle-related diseases account for a significant health burden in the region, including cardiovascular disease, which is now the leading cause of death and disability among adults.^19^ The average population age is also rising, leading to an elevated chronic disease burden and a heightened demand on national health systems.^20^ Thus, the focus of health care resource allocation has shifted from communicable disease towards non-communicable, lifestyle-related disease in recent decades and the chronic management of these conditions.

The consequence of the emergence of cardiovascular disease and risk factors, coupled with an ageing population, is that the development of AF is more likely and the prognosis of AF substantially worse.^19^ Estimates suggest that over 50% of patients with AF in Latin America have arterial hypertension, while up to 40% of patients have concurrent heart failure or diabetes at the time of diagnosis.^19^ For many, hypertension is poorly controlled, particularly in older, less educated and obese patients.^20^ Furthermore, the metabolic syndrome has become common in Latin America and is strongly associated with the development of AF and the risk of future stroke.^21^ These risk factors not only impact the prognosis of AF, but also predispose to future cardiovascular events, including ischemic stroke.^22,23^ As a result, a rising number of stroke deaths have been recorded in the region, which is estimated to increase over the next few decades.^24^

Latin American data on the characteristics of stroke patients are limited, but some data demonstrate that there is a relatively higher rate of hemorrhagic stroke compared to high-income nations (26% vs. 9%).^25^ The primary etiology of stroke is less frequently identified compared to Western nations, highlighting the challenges to patient investigation in the region.^26^ This is evidenced by a low rate of vascular imaging for stroke investigation compared to Western nations (20% versus 77%).^25^ Stroke risk has been associated with smoking and self-reported hypertension,^26^ both of which are common in Latin American populations.^20^

### Challenges to AF management in Latin America

Thromboprophylaxis in AF has traditionally been facilitated through the use of either VKAs (eg, warfarin) or aspirin in Latin America.^27^ A recent analysis of anticoagulant use in seven Latin American nations found that 66-75.8% of patients with AF receive VKA or VKA plus aspirin, while the remainder receive either no treatment (18.3-24.6%) or triple therapy with VKA, aspirin and an additional antiplatelet agent (3.8-9.6%) (Table 1).^19^ Even more concerning are recent figures from a Brazilian primary care database in which only 1.5% of patients were found to be on VKA therapy.^28^ These figures reflect the reliance on warfarin and aspirin in the region, but also highlight the missed opportunities in stroke prevention for the AF patients who do not receive any anticoagulants.

**Table 1:** Outpatient management of atrial fibrillation in seven Latin America nations. Adapted from (19)

	**Argentina**	**Brazil**	**Chile**	**Colombia**	**Mexico**	**Peru**	**Venezuela**
No treatment	24.6%	18.3%	21.2%	22.4%	22.5%	21.4%	20.3%
VKA	53.4%	58.2%	52.3%	45.2%	56.2%	56.2%	57.5%
VKA+Aspirin	12.6%	17.6%	20.3%	28.6%	16.6%	15.7%	12.6%
VKA + Aspirin + antiplatelet	9.4%	5.9%	6.2%	3.8%	4.7%	6.7%	9.6%

VKA: vitamin K antagonist.

There have been notable challenges with the use of both warfarin and aspirin in practice, contributing to the rising stroke prevalence in the region. Warfarin use demands careful anticoagulant monitoring, which may not be possible in many parts of Latin America, or may not be practicable due to patient access to health services or service cost.^27^ Data from the ROCKET-AF study found that the median TTR achieved in patients with AF managed with warfarin in Latin American countries is generally lower than that seen in Western Europe and the US, with a median TTR of 59%.^29^ This is lower than the recommended 70% as set out in existing Western guidelines, such as the American College of Chest Physicians guidelines and the European Society of Cardiology guidelines.^15,16^ One study has shown that 21.4% of patients consider periodic blood tests to be significant barriers to oral anticoagulant adherence in Brazil, while adherence to VKA to achieve optimal TTR only occurred in 54% of patients.^30^ Similar data has been obtained in a separate study, where analysis of 127 outpatients demonstrated VKA (phenprocoumon) use only achieved optimal TTR in 60.7% of patients.^31^ Therefore, achievement of optimal TTR with warfarin may be lacking in Latin America, leading to poorer outcomes.

### AF in elderly patients

It is also recognized that physicians in Latin America often feel that the risk of bleeding is elevated in older patients with AF, which may lead to physicians choosing to withhold oral anticoagulant therapy,^4^ despite the general consensus in Western literature that the benefits of anticoagulation generally outweigh falls risk.^15,16^ However, one study from Argentina found that of 840 patients with AF and a high risk of stroke, only 48.5% were managed with oral anticoagulants (predominantly warfarin), while only 17.1% of patients had any clear contraindications to warfarin therapy.^32^ Similarly, a chart review of 301 patients in a Brazilian hospital found that only 46.5% of patients with AF received anticoagulation therapy in-hospital and 57.8% during one year following initial therapy or in the outpatient population.^33^ Therefore, warfarin may be underused in the Latin American context, leading to suboptimal thromboprophylaxis, particularly in elderly patients.

The combined use of warfarin and aspirin in the Latin American population forms a significant proportion of patients receiving thromboprophylaxis.^19^ Current guidelines suggest that monotherapy with a VKA should be preferred in the majority of patients with AF, due to the increased risk of bleeding associated with combined use of VKA and aspirin, although combined regimens may be useful in the AF patient presenting with an acute coronary syndrome and/or undergoing percutaneous coronary intervention or stenting.^34-36^ Data from Latin America are scarce with regards to the decision-making processes underlying the relatively low use of monotherapy in this population, but it is apparent that the use of aspirin is more common than in Western nations.^28^ This is a concern, as aspirin elevates the risk of bleeding, especially when used in combination with oral anticoagulants.^37^ The net clinical benefit (NCB) for aspirin considering stroke and mortality reduction against serious bleeding is neutral or negative (depending on NCB definition), even with patients with a single stroke risk factor.^38^

### Valvular disease and Chagas in Latin America

Latin America has a significant burden of valvular heart disease associated with AF, particularly in the elderly population. It has been shown that up to 60% of valve disease is associated with rheumatic fever and that around half of patients with valvular disease present with AF.^39^ Hence, this is an important subgroup in Latin America, which may pose specific challenges to physicians. Systemic anticoagulation is generally favoured in all patients without contraindications with mitral valve disease and AF due to the high risk of stroke and mortality.^40^

Chagas disease is also a significant public health issue in Latin America, with recent data from Brazil highlighting the burden of cardiac disease associated with the condition. Self-reported Chagas disease was associated with electrocardiographic abnormalities in over two-thirds of patients in one analysis, with 5.4% of patients demonstrating AF.^41^ A recent review of Brazilian primary care patients suggested that Chagas disease was present in 2.9% of patients with AF, with ECG abnormalities strongly predicting the development of AF and adverse outcomes.^41^ Therefore, these patients are considered a high-risk group for the development of AF.

The contemporary picture in Latin America is that thromboprophylaxis is suboptimal in patients with AF. Many ambulatory patients with significant risk factors for future stroke do not receive anticoagulant therapy and may be receiving inappropriate therapy with combined regimens or aspirin alone. The potential for the NOAC class of drugs to fill this therapeutic void will be explored in the following section.

### NOACs for thromboprophylaxis in patients with non-valvular AF

The NOACs have revolutionized the potential to prevent stroke in patients with non-valvular AF and have been endorsed as a major treatment option by international bodies, including the European Society of Cardiology.^34^ Two classes of NOACs are currently available, with four drugs licensed for use as anticoagulants in patients with non-valvular AF: direct thrombin inhibitors (dabigatran) and direct factor Xa inhibitors (rivaroxaban, apixaban and edoxaban). Each drug has a distinct dosing profile and set of contraindications (Table 2).

**Table 2:** Non-vitamin K antagonist oral anticoagulant dosing recommendations

	**Dabigatran**	**Rivaroxaban**	**Apixaban**	**Edoxaban**
Licensed dose for stroke prevention in AF	150 mg twice daily	20 mg once daily	5 mg twice daily	60 mg once daily
**Renal dose modification**				
CrCl > 50 ml/min	No dose modification	No dose modification	No dose modification	No dose modification^a^
CrCl 30–49 ml/min	Consider 110 mg twice daily	15 mg once daily	No dose modification	30 mg once daily
CrCl 15–29 ml/min	Not recommended	15 mg once daily	2.5 mg twice daily	30 mg once daily
CrCl < 15 ml/min	Not recommended	Not recommended	Not recommended	Not recommended
Cautions	Age > 80 years Weight < 60kg Macrolide antibiotics Amiodarone	Age > 80 years Weight < 60kg Macrolide antibiotics Carbamazepine Phenytoin	Age > 80 years Weight < 60kg Diltiazem	Concomitant use of P-glycoprotein inhibitors or Weight < 60kg = adjust dose to 30 mg once daily
Medications contraindicated in conjunction with NOAC	Ketoconazole Itraconazole Carbamazepine Phenytoin Dronedarone	Ketoconazole Itraconazole	Ketoconazole Itraconazole Carbamazepine Phenytoin	Rifampin

AF: atrial fibrillation; NOAC: non-VKA oral anticoagulant.

a Edoxaban is not recommended to be used in patients with creatinine clearance > 95 mL/min due to the increased risk of ischemic stroke compared with warfarin.

#### Dabigatran

Dabigatran is a direct thrombin inhibitor licensed for use by both the US Food and Drug Administration (FDA) and the European Medicines Agency (EMA).^42^ Phase III trial data is based on the international, multicenter, randomized RE-LY (Randomized Evaluation of Long-Term Anticoagulation Therapy) trial, which analyzed 18,113 AF patients with a mean CHADS_2_ score of 2.1.^43^ Patients were randomized to two different doses of dabigatran (110 mg or 150 mg twice daily) or received warfarin dose-adjusted to achieve an International Normalized Ratio (INR) of 2-3, with a mean TTR of 64%. At two-years follow-up, dabigatran 150 mg twice daily was found to be superior to warfarin in the prevention of stroke and systemic embolism [Relative risk (RR) 0.66, 95% confidence interval (CI) 0.53-0.82, p < 0.001], while the lower dose of dabigatran was non-inferior to warfarin. It was also found that the risk of hemorrhagic stroke was significantly lower with both doses compared to warfarin (110 mg: RR 0.31, 95% CI 0.17-0.56, p < 0.001; 150 mg: RR 0.26, 95% CI 0.14-0.49, p < 0.001). With respect to mortality, vascular and non-hemorrhagic major events, the advantages of dabigatran 150 mg twice daily were greater at study sites where the TTR was lowest in the warfarin arm.^43^ Geographical sub-group analysis of the Latin American cohort of the RE-LY study^44^ found that this cohort experienced similar results as the main group. This highlights the importance of TTR when comparing warfarin and NOACs.

The safety profile of dabigatran was dose-dependent, with a lower prevalence of major bleeding with 110 mg twice-daily dabigatran (RR 0.80, 95% CI 0.69-0.93, p = 0.003) but not with patients on 150mg twice daily when compared to warfarin (RR 0.93, 95% CI 0.81-1.07, p = 0.31). The risk of intracranial bleeding and life-threatening bleeding was lower with dabigatran versus warfarin, although there was an increased likelihood of gastrointestinal bleeding with dabigatran 150mg twice daily.^43^

#### Rivaroxaban

Rivaroxaban is a direct factor Xa inhibitor that has been approved by both the FDA^45^ and the EMA.^46^ The ROCKET-AF (Rivaroxaban Versus Warfarin in Nonvalvular AF) multicenter, randomized controlled trial analyzed 14,264 AF patients with CHADS_2_ ≥ 2 (mean = 3.47; high stroke risk).^47^ Patients were randomized to either rivaroxaban 20 mg once daily (15 mg if Creatinine clearance was 30-49 mL/min) or warfarin with a target INR of 2-3 (median TTR 58%). The median follow-up period was 1.9 years at which rivaroxaban was shown to be non-inferior to warfarin for stroke and systemic embolism prevention [Hazard ratio (HR) 0.88, 95% CI 0.74-1.03]. There was a lower rate of intracranial hemorrhage and fatal hemorrhage with rivaroxaban compared to warfarin. However, deaths due to ischemic stroke were comparable between treatment arms, and gastrointestinal bleeding was more likely with rivaroxaban compared to warfarin (3.2% vs. 2.2%, p < 0.001).

Supplementary data from the ROCKET-AF study suggests that there are no major differences in stroke occurrence, safety or major bleeding between warfarin and rivaroxaban use in the Latin American cohort of the study, although there is a trend towards less major bleeding with rivaroxaban.^47^ The proposed XANTUS-EL (Xarelto for Prevention of Stroke in Patients with Nonvalvular Atrial Fibrillation, Eastern Europe, Middle East, Africa and Latin America) study,^48^ a prospective, observational post-authorization, non-interventional study is designed to analyze the safety and efficacy of rivaroxaban in routine clinical use in Eastern Europe, the Middle East, Africa and Latin America. The results of this study should provide clarification of the role of rivaroxaban as stroke prophylaxis in patients with AF in Latin America.

#### Apixaban

Apixaban is a direct factor Xa inhibitor approved for use in AF thromboprophylaxis by the FDA^49^ and EMA.^50^ Efficacy and safety data for apixaban versus warfarin were analyzed in the ARISTOLE multicenter, international, randomized trial.^51^ Patients (n = 18,201) with non-valvular AF and CHADS_2_ ≥ 1 (mean = 2.1) were randomized to either apixaban 5 mg twice daily or warfarin with a target INR 2-3 (median TTR 66%) and followed up for a median of 1.8 years. It was demonstrated that apixaban was superior to warfarin for stroke and systemic embolism prevention (1.27% vs. 1.60%, HR 0.79, 95% CI 0.66-0.95, p = 0.01). Apixaban was also associated with a reduction in hemorrhagic stroke (0.24% vs. 0.47%, HR 0.51, 95% CI 0.35-0.75, p < 0.001) and all-cause mortality (3.52% vs. 3.94%, HR 0.89, 95% CI 0.80-0.99, p = 0.047), compared to warfarin. The safety profile of apixaban was favorable compared to warfarin, with a lower rate of major hemorrhage (2.13% vs. 3.09%, HR 0.69, 95% CI 0.60-0.80, p < 0.001) and comparable occurrences of gastrointestinal bleeding (0.76% vs. 0.86%, HR 0.89, 95% CI 0.70-1.15). Bleeding in the Latin American cohort of this study (n = 3,460) was also lower with apixaban than warfarin (2.1% vs. 3.5%), which may be associated with either poor TTR control in the Latin American cohort or may be a genuine effect of apixaban. Further subgroup analysis of the Latin American cohort suggests that results in terms of safety and efficacy are consistent with those seen in total study population, with minimal geographic variability within the region.^52^

Apixaban was also compared to aspirin for use in AF thromboprophylaxis in the AVERROES (Apixaban Versus Acetylsalicylic Acid (ASA) to Prevent Stroke in Atrial Fibrillation Patients Who Have Failed or are Unsuitable for Vitamin K Antagonist Treatment) trial.^53^ Patients who failed or were not suitable for VKA therapy (n = 5,599) were included in the study and were randomized to either apixaban 5 mg twice daily or aspirin 81-324 mg daily. The study was terminated prematurely due to the overwhelming superiority of apixaban, with a 55% reduction in stroke or systemic embolism.^53^ Major bleeding or intracranial bleeding was not significantly different between apixaban and aspirin.

#### Edoxaban

Edoxaban is the most recent NOAC to gain approval by the FDA in the US and is approved for use by the Japanese Ministry of Health.^54^ A large, international, randomized controlled trial, ENGAGE AF-TIMI 48 (Effective Anticoagulation with Factor Xa Next Generation in Atrial Fibrillation - Thrombolysis in Myocardial Infarction study 48), was conducted to explore the outcomes in 21,105 AF patients with CHADS_2_ score ≥ 2.^55^ The study was based on an initial phase 2 trial which identified two doses of edoxaban that had comparable levels of bleeding risk to warfarin (30 mg or 60 mg once daily).^56^ Patients were randomized to either high dose (60 mg or 30 mg dose-reduced) or low dose (30 mg or 15 mg dose-reduced) regimens, or warfarin dose-adjusted to achieve an INR of 2-3, with a median TTR of 68%. The median follow-up was 2.8 years, during which time 25.3% of patients randomized to edoxaban underwent dose reduction based on specific risk factors known to increase drug exposure (Creatinine clearance 30-50mL/min, concurrent use of verapamil or quinidine, or weight ≤ 60 kg).

Modified intention-to-treat analyses were performed, demonstrating that both doses of edoxaban were non-inferior to warfarin in the prevention of stroke and systemic embolism: higher dose 1.18% vs. 1.50%, hazard ratio (HR) 0.79, 97.5% CI 0.63-0.99, p < 0.001; lower dose 1.61% vs. 1.50%, HR 1.07, 97.5% CI 0.87-1.31, p = 0.005. In the intention-to-treat population there was a trend towards superiority for the high-dose edoxaban, although this was not statistically significant (HR 0.87, 97.5% CI 0.73-1.04, p = 0.08). There was a significant reduction in the occurrence of hemorrhagic stroke when comparing the high-dose edoxaban regimen to warfarin, as seen with other NOACs (0.26% vs. 0.47%, HR 0.54, 95% CI 0.38-0.77, p < 0.001). Overall, there were significant benefits seen with higher-dose edoxaban versus warfarin regarding the prevention of stroke and systemic embolism and a composite endpoint of stroke, systemic embolism or cardiovascular death (HR 0.87, 95% CI 0.78-0.96, p = 0.005).

The safety profile of edoxaban versus warfarin was also considered to be favorable, with a reduction in major hemorrhage (high-dose 2.75% vs. 3.43%, HR 0.80, 95% CI 0.71-0.91, p < 0.001; low-dose 1.61% vs. 3.43%, HR 0.47, 95% CI 0.41-0.55, p < 0.001), extra-cranial bleeding and non-major bleeding. It should be noted however, that gastrointestinal bleeding was observed more frequently in the high-dose edoxaban regimen, compared to low-dose edoxaban and warfarin (1.51%, 0.82% and 1.23%, respectively).^55^ Therefore, edoxaban has proven efficacy in stroke and systemic embolism prevention in comparison with warfarin, while bleeding events may be dose-dependent.

In an analysis of the Latin American cohort of this study (n = 2,661),^55^ the primary efficacy of prevention of stroke or systemic embolism was consistent with the overall population for both lower and higher dose edoxaban compared to warfarin (30 mg dose 2.15% vs. 2.50%; 60 mg dose 1.61% vs. 2.50%, p = 0.32). The trend towards edoxaban being a superior agent was stronger than as seen in Western European patients, though this did not approach statistical significance. Additionally, the safety profile demonstrated a trend toward favoring edoxaban vs. warfarin in this cohort, with both lower dose edoxaban (1.66% vs. 3.74%, p = 0.50) and higher dose edoxaban (2.65% vs. 3.74%, p = 0.35) versus warfarin therapy.^55^ The analyses were comparable to the results seen in the general population for both safety and efficacy in the Latin American cohort.

Recent modeling analyses have also been published, providing an estimate of the NCB of edoxaban versus no treatment, aspirin, aspirin and clopidogrel, or warfarin.^57-59^ It was shown that edoxaban 60 mg has a NCB of 8.9 events saved per 1000 patients compared to warfarin therapy or no treatment, an estimated prevention of 30,300 thromboembolic events, major bleeds and deaths annually in European AF patients.^57^ Indeed, both edoxaban 30 mg and 60 mg were found to have a favourable NCB compared to warfarin, with the level of benefit relating directly to CHADS_2_, CHA_2_DS_2_-VASc and HAS-BLED scores; patients with CHA_2_DS_2_-VASc ≥ 2 benefitted from both doses of edoxaban versus warfarin, although the 60 mg dose was associated with the greater NCB overall.^58^ Hence, these modelling studies highlight the potential NCB of edoxaban compared with existing anticoagulation approaches in practice, although demonstration of these effects in the Latin American population remains to be seen.

#### Meta-analyses of NOACs

On an individual trial basis the NOACs have been shown to be non-inferior to warfarin for the prevention of strokes in patients with AF. Large meta-analyses have been conducted of these trials, demonstrating the efficacy of NOACs compared to warfarin.^60,61^ The NOACs have been associated with a reduced risk of systemic embolism and stroke compared to warfarin (RR 0.81, 95% CI 0.73-0.91, p < 0.0001), a reduced risk of intracranial hemorrhage (RR 0.48, 95% CI 0.39-0.59, p < 0.0001) and a reduction in all-cause mortality (RR 0.90, 95% CI 0.85-0.95, p = 0.0003).^60^ However, these agents have been associated with an increased risk of gastrointestinal bleeding (RR 1.25, 95% CI 1.01-1.55, p = 0.04). It is worth noting that the most significant benefits of NOACs were observed in centers where the time in therapeutic range (TTR), defined as maintenance of the INR between 2.0-3.0, was less than 66%, indicating that NOACs have benefits where control of anticoagulation is suboptimal.^60^

Data for the use of direct thrombin inhibitors, including the NOAC dabigatran, are available in several meta-analyses. A Cochrane review found that direct thrombin inhibitors were generally comparable to warfarin for many outcomes, although dabigatran 150 mg twice daily was associated with significantly fewer vascular deaths and ischemic events compared to warfarin [Odds ratio (OR) 0.86, 95% CI 0.75-0.99).^61^ Direct thrombin inhibitors were also associated with significantly fewer major hemorrhagic events, including hemorrhagic strokes, compared to warfarin. Similarly, a Cochrane meta-analysis of direct factor Xa inhibitors found that these agents were associated with a significant reduction in ischemic and hemorrhagic strokes (OR 0.78, 95% CI 0.69-0.89) and a reduction in major bleeding episodes (OR 0.89, 95% CI 0.81-0.98) compared to warfarin therapy.^62^ A recent meta-analysis has confirmed the low bleeding risk associated with apixaban both for all-cause bleeding (RR 0.60, 95% CI 0.40-0.88) and intracranial bleeding (RR 0.89, 95% CI 0.81- 0.99) compared to warfarin.^49^

Although data provide a strong basis for the use of NOACs in stroke prevention in patients with AF, there is currently no data to suggest that one particular NOAC may be superior to the others. Indirect comparisons have limited value in decision-making, due to the differences in trial design and characteristics,^63^ and therefore evaluation of the caveats of individual agents is needed when selecting a drug for a specific patient profile.^35^ The following section will consider how patient profiles can be used to inform clinical decision-making in this context and how these may relate to the Latin American AF population.

### Guidelines in Latin America

A number of guidelines specific to Latin America have been devised for the management of patients with AF and the use of anticoagulation therapy therein. The Brazilian Society of Cardiac Arrhythmias and the Brazilian Cardiogeriatrics Society guidelines are two of the most commonly used resources for physicians in the region.^4,64^ These guidelines emphasize the importance of rhythm and rate control in AF patients and focus on the role of warfarin as the main oral anticoagulant in patients who have undergone a thorough bleeding risk assessment and stroke risk assessment. The specific role of NOACs in the published guidelines is not described, in contrast with European and other national guidelines, although the guidelines are similar in terms of patient risk stratification and the general stages involved in AF management and stroke prevention.^15,16,30^ However, one key difference is use of the CHADS_2_ score in Brazilian guidelines, compared to the use of the CHA_2_DS_2_-VASc score in guidelines from Europe and North America.^16,35^

The use of VKA therapy is prioritized in Brazilian guidelines, while aspirin use is a major feature in patients with lower risk of stroke (Table 3). No specific TTR recommendations are set out in these guidelines, although optimization of TTR > 70% maximizes the effects of VKA therapy and is recommended in guidelines outside of Latin America.^35^ The potential for the use of NOACs in systems where warfarin monitoring may be problematic has been highlighted in an updated analysis of contemporary guidelines in Brazil.^64^

**Table 3:** Brazilian guidelines for the use of anticoagulants in the prevention of stroke in patients with atrial fibrillation. Adapted from (64)

**Stroke risk stratification**	**Therapy**
**High risk**	
Prior thromboembolism Rheumatic mitral stenosis >1 of: aged >75 years, hypertension, heart failure, impaired left ventricular systolic function, type 2 diabetes	Oral VKA (INR 2–3)
**Moderate risk**	
1 of: aged >75 years, hypertension, heart failure, impaired left ventricular systolic function, type 2 diabetes	Oral VKA (INR 2–3) Or Aspirin 81–325 md daily
**Low risk**	
No other risk factors	Aspirin 81–325 mg daily

INR: International Normalized Ratio; VKA: vitamin K antagonist.

Despite the existence of guidelines, there is evidence that available recommendations are not adhered to in practice, potentially limiting the ability to optimize patient care.^65^ Physicians with over 25 years of experience tend to have less awareness of guidelines, or are more likely to disagree with existing guidelines, with only 71.8% favoring VKA as an initial anticoagulant in AF, as recommended in guidelines, compared to over 90% of more junior cardiologists.^65^ Furthermore, this survey found that 10% of long practicing cardiologists in Brazil do not adhere to any AF guidelines, while a significant number do not routinely apply risk scores before initiating anticoagulation therapy.^65^ A review of prescribing habits in a Brazilian cardiology department found that anticoagulant use according to the Brazilian guidelines was 55%, which was consistent with international guidelines, while 86% of patients with a high risk of embolism were prescribed oral anticoagulants.^66^ These statistics suggest that guidelines may not be adhered to strictly in practice, with a significant proportion of Latin American patients not receiving adequate anticoagulation.

### Stroke risk stratification and treatment selection

The determination of stroke risk for an individual patient is a multifactorial process, dependent on a number of distinct risk factors. The CHA_2_DS_2_-VASc (Congestive heart failure, Hypertension, Age ≥ 75, Age between 65 and 74, Diabetes mellitus, prior Stroke, TIA or thromboembolism, VAscular disease, Sex female) score is commonly used to predict stroke risk and potential utility of anticoagulation therapy (Table 4).^67^ A score of 0 (males) or 1 (females) suggests low-risk of stroke and in these patients the risks of anticoagulation are likely to outweigh the benefits, while higher scores should prompt an assessment of bleeding risk and initiation of anticoagulation.^67^ The CHA_2_DS_2_-VASc score was initially derived in predominantly White European populations, but it has subsequently been validated in other ethnic populations.^68^ Specific validation in Latin America has not been conducted to date. However, the CHA_2_DS_2_-VASc score has been shown to be superior to the CHADS_2_ score in defining low and intermediate risk populations that are unlikely to benefit from anticoagulation.^69^ This suggests that patients in Latin America may benefit from the use of the CHA_2_DS_2_-VASc score rather than CHADS_2_, as seen in contemporary guidelines in the region.^3,64^ The development of a Latin America-specific scoring system has been proposed by one group, based on factors including age, National Institute of Health Stroke Scores and the presence of left atrial enlargement.^70^ However, this will need further validation in future studies before it can be applied to the Latin American population.

**Table 4:** CHA2DS2-VASc risk assessment scoring for stroke risk in patients with atrial fibrillation. Adapted from (68)

**Definition**	**Score**	**Notes**
Congestive Heart Failure	1	Moderate-to-severe systolic left ventricular dysfunction
Hypertension	1	Patient on antihypertensives or two concurrent readings >140 mmHg systolic and/or > 90 mmHg diastolic
Age >75 years	2	-
Diabetes mellitus	1	On antihyperglycaemic drugs of fasting blood glucose >7 mmol/L
Stroke/TIA/thromboembolism	2	-
Vascular disease (Prior MI, PAD, aortic plaque)	1	Eg, prior MI, angina, intermittent claudication, thrombosis, previous surgery on abdominal aorta.
Age 65-74 years	1	-
Female sex	1	-

TIA: transient ischemic attack; MI: myocardial infarction; PAD: peripheral artery disease.

The assessment of bleeding risk can be performed using a number of different scores, including the ATRIA (Anticoagulation and Risk Factors in Atrial Fibrillation), HEMORR_2_HAGES [Hepatic or renal disease, Ethanol abuse, Malignancy, Older (age > 75 years), Reduced platelet count or function, Hypertension (uncontrolled), Anemia, Genetic factors, Excessive fall risk, and Stroke], and HAS-BLED [Hypertension, Abnormal renal or liver function, Stroke, Bleeding, Labile INRs, Elderly (age > 65 years) and Drugs (alcohol)] scores.^71,72^ The HAS-BLED score has been shown to have the best predictive value for bleeding risk.^73^ Under-use of anticoagulation, based on a perceived risk of hemorrhage remains common.^74^ However, a high HAS-BLED score is not an excuse to withhold anticoagulation, as the net clinical benefit balancing ischemic stroke reduction against serious bleeding is even greater in such patients.^72^ A high HAS-BLED score is an indicator to 'flag up' the patient for more careful review and follow-up, and to address the potentially correctable bleeding risk factors, such as uncontrolled hypertension, labile INRs, concomitant use of aspirin and NSAIDs with anticoagulation.

**Figure 1:**
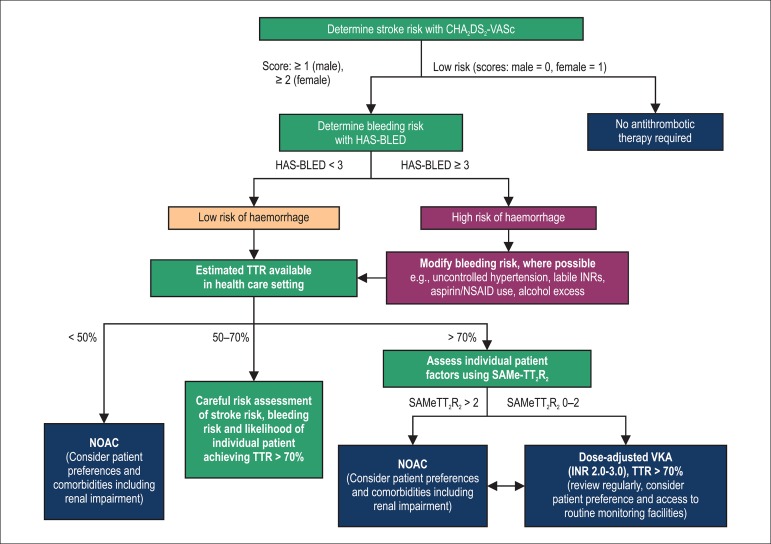
Algorithm for anticoagulation in Latin American patients with atrial fibrillation. The decision to initiate anticoagulant therapy is based on the use the CHA *_2_* DS *_2_* VASc, HAS-BLED and SAMeTT2R2 scores through determination of stroke risk, bleeding risk and likelihood of warfarin success, respectively. INR: international normalised ratio; NOAC: non-vitamin K antagonist oral anticoagulants; NSAID: non-steroidal anti-inflammatory drug; TTR: time in therapeutic range; VKA: vitamin K antagonist. Adapted from (81).

The decision to use warfarin or NOACs in the management of patients with a high risk of stroke requires an appreciation of time in therapeutic range (TTR) for the individual patient.^75^ Patients who fall outside of TTR more frequently are less likely to benefit from warfarin therapy and may be at an increased risk of stroke.^76^ Although the reasons for poor control of anticoagulation levels may be varied, the National Institute for Health and Care Excellence (NICE) recommends that patients with TTR < 65% should be re-assessed.^35^ Where patient compliance is not a factor and TTR cannot be adequately maintained, the use of NOACs is likely to provide more significant clinical benefits.^38^ The SAMe-TT_2_R_2_ score has been devised and validated in Europe as a means of predicting poor control of warfarin therapy in patients with AF (Table 5).^77^ The SAMe-TT_2_R_2_ aids decision making whereby a patient with a SAMe-TT_2_R_2_ score of 0-2 is likely to do well on a VKA with a high TTR, whilst those with a SAMe-TT_2_R_2_ score of > 2 are less likely to achieve a optimal TTR, and a NOAC would be a better option.^78^ Thus, a 'trial of warfarin' can be avoided, given that some patients would be exposed to a high risk of ischemic stroke during the inception phase of warfarin therapy.^79^ The SAMe-TT_2_R_2_ score has been related to labile INRs, and consequently, more bleeding, thromboembolism and death.^80^ The high incidence of comorbidities and smoking in the Latin American AF patient population would suggest that a high score would be achieved, predicting poor warfarin control and favoring the use of NOACs.^77^ A suggested algorithm for the use of scoring systems in the determination of stroke risk, bleeding risk and the likelihood of VKA effectiveness is demonstrated in Figure 1.

**Table 5:** SAMe-TT2R2 score. Adapted from (75)

**Acronym**	**Definitions**	**Score**
S	Sex (female)	1
A	Age (< 60 years)	1
Me	Medical history: 2 or more of hypertension, diabetes, CAD/MI, PAD, CHF, previous stroke, pulmonary disease, and hepatic or renal disease.	1
T	Treatment (interacting drugs eg, amiodarone)	2
T	Tobacco use (within 2 years)	2
R	Race (non-White)	8

CAD: coronary artery disease; CHF: congestive heart failure; MI: myocardial infarction; PAD: peripheral artery disease.

### Patient profiling and NOAC selection

**Figure 2:**
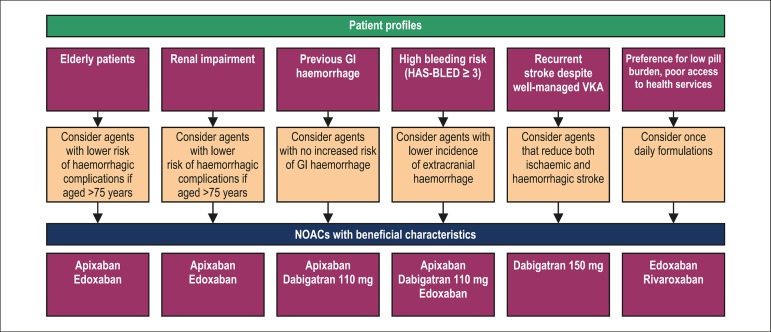
Patient profiling in NOAC selection. The patient groups highlighted are likely to be of greatest importance to the Latin American context. Individual non-VKA oral anticoagulant (NOAC) use is based on non-inferiority to warfarin for stroke prevention in non-valvular atrial fibrillation and individual drug characteristics. Adapted from (81). VKA: vitamin K antagonist; GI: gastrointestinal.

A number of patient profiles have been identified in the context of stroke prevention in AF, which may influence the choice of NOAC based on the potential for complications versus the potential for efficacy (Figure 2). These profiles have been reviewed in detail elsewhere.^81^ However, the identification of these patient profiles in Latin American AF patients has yet to be performed. Patient profiling based on pharmacogenetic techniques has been reported within the context of anticoagulant selection in Brazilian patients^82^ highlighting the importance of considering European/African ancestry among the Latin American population. However, prospective studies of dosing algorithms based on these factors are lacking at present. Therefore, it is worth considering the clinical implications of patient profiles that are likely to be most common in Latin America in determining the selection of NOACs in this region, including elderly patients, patient with renal impairment and those at-risk of bleeding events.^81^

Elderly patients form the majority of patients with AF in Latin America, with over 70% of AF patients aged 60 years or older.^19^ These patients are at an increased risk of stroke compared to younger patients due to an increased risk of bleeding with age.^34^ However, it should also be considered that many patients are at an increased risk of falls and subsequent hemorrhage, potentially limiting the use of anticoagulation in this population.^80^ Consequently, it is considered prudent to select NOACs that are less likely to be associated with hemorrhage in the elderly, including apixaban and edoxaban.^83^ However, elderly patients form a heterogeneous group and therefore additional risk factors and profiles may have a greater impact on the selection of NOAC.

In addition to elderly patients, patients with comorbid renal impairment may be at a higher risk of hemorrhagic complications during anticoagulant therapy.^84^ The contemporary prevalence of renal impairment in Latin America is largely unknown, although data suggests that increasing rates of type 2 diabetes have been associated with a rise in end-stage renal failure, indicative of rising rates of renal impairment in the population.^84^ Renal impairment is associated with poor control of INR and a worse outcome and therefore adversely affects the use of VKA therapy.^85^ Therefore, patients with renal impairment, defined as an estimated glomerular filtration rate ≤ 80 mL/min, may benefit from the use of apixaban, which has been shown to be more effective than warfarin in stroke prevention, regardless of renal function, and is not associated with hemorrhagic complications, as reported with dabigatran.^86^ Edoxaban has also demonstrated some promising results in patients with renal impairment, with low bleeding and adverse event rates.^87^

Other important patient characteristics to consider when choosing a NOAC include a previous history of gastrointestinal hemorrhage, a high bleeding risk (HAS-BLED ≥3) and those with recurrent stroke, despite optimal warfarin management.^81^ For all of these high risk patients a NOAC with a low risk of hemorrhage would potentially be beneficial (eg, apixaban or dabigatran), with dabigatran 150 mg having been shown to reduce the risk of both intracranial bleeding and hemorrhagic stroke.^88^ It should be noted that edoxaban, dabigatran and rivaroxaban have been associated with an increased risk of gastrointestinal bleeding when higher dose regimens are used.^45,49,60^

Patient values and preference should also be considered when prescribing anticoagulant therapy, as the dosing schedule and side effect profile of a drug may determine adherence and efficacy^89^. Poor adherence is more likely to be associated with suboptimal clinical benefits with NOAC therapy, due to the relatively short half-life of these agents compared to warfarin. Once-daily dosing is available with rivaroxaban and edoxaban, which may be preferable to twice daily dosing in some patients^90^.

### Decision-making in Latin America

The use of patient profiles can be helpful in selecting the most appropriate course of anticoagulant therapy, where we can fit the drug to the patient and vice versa (Figure 2). However, due to the lack of data available in the Latin American context, additional considerations may need to be made when prescribing these agents. One of the main challenges to stroke prevention in the region is the large socioeconomic disparity in health outcomes and access to health care.^19^ Patients with low income, low education, and those living in rural communities have reduced access to health care services, leading to poorer outcomes for many chronic diseases.^91^ Hence, the challenge of maintaining anticoagulation in this group may relate not only to the ability for patients to accurately adhere to their medication schedule, but also their ability to attend for monitoring and follow-up appointments.

One of the main advantages of the NOACs compared to warfarin is that they do not require routine anticoagulant monitoring. This is an appealing prospect in many regions of Latin America, where inadequate levels of warfarin monitoring may limit the use of this drug in practice.^92^ Furthermore, the cost-effectiveness of NOAC use is largely unclear in the region and may be a significant factor limiting the use of these agents in practice. One analysis from Costa Rica utilized a decision-tree model to compare the cost-effectiveness of apixaban, rivaroxaban, dabigatran and warfarin for the prevention of stroke and bleeding, and found that apixaban was the most cost-effective option.^93^ This was largely due to the perceived efficacy of the NOAC class of agents and the low associated risk of hemorrhage, in additional to the associated resource costs of warfarin monitoring. Further analyses from Argentina^94^ and Venezuela^95^ suggest that apixaban is a cost-effective alternative to warfarin therapy due to the reduction in stroke and bleeding events. However, recent data suggest that many cardiovascular disease medications remain unaffordable for many nations in Latin America, particularly among poorer communities.^96^ Therefore, the potential for NOACs to be a cost-effective option in Latin America exists, although further studies will be needed to confirm this finding.

Another factor that has been associated with poor use of anticoagulation in patients with AF at risk of stroke is the status of the prescribing physician. It has been shown that patients who have attended tertiary centers and have access to cardiologists are more likely to be prescribed appropriate anticoagulation compared to physicians of other specialties.^97^ This may be due to the difficulty of returning to the same treatment center for monitoring and treatment.^97^ However, educating non-specialist physicians regarding the benefits of anticoagulation may be another factor that could improve the use of anticoagulation.

The use of NOACs, which do not require monitoring, may overcome many of the difficulties seen with anticoagulation in Latin America. However, the lack of these drugs within the public health system of many nations is a significant obstacle to their widespread use.^97^ There has been a study showing the potential benefits of apixaban in Latin America, suggesting the use of NOACs in this region may be favorable due to perceived benefits in practice compared to VKA therapy.^98^ The more urgent issue that needs to be addressed in the region is the recognition of the value of anticoagulation with VKAs or NOACs in the prevention of stroke, as many physicians do not prescribe these agents appropriately. Therefore, increasing awareness of the need for anticoagulation in patients with AF should be a public health priority in Latin America, facilitating the development of more robust anticoagulation services.

## Conclusion

The NOACs represent a significant advancement in the potential to prevent stroke in patients with non-valvular AF. Evidence from large, randomized studies and subsequent meta-analyses demonstrates the non-inferiority of NOACs compared to warfarin in Western populations, although more data is needed on Latin American cohorts. Additional benefits compared to warfarin are likely to include the lack of need for routine anticoagulant monitoring, reduced drug and food interactions and the predictability of the pharmacokinetic activity of the drug. However, the decision to use NOACs instead of warfarin, and the selection of which NOAC to use, remains a complex process based on individual patient characteristics. Identifying how these characteristics interact with wider health processes and systems in Latin America will be a future challenge for physicians in the region.
